# Chemotherapy-Induced Molecular Changes in Skeletal Muscle

**DOI:** 10.3390/biomedicines11030905

**Published:** 2023-03-15

**Authors:** Mafalda Barbosa Pedrosa, Samuel Barbosa, Rui Vitorino, Rita Ferreira, Daniel Moreira-Gonçalves, Lúcio Lara Santos

**Affiliations:** 1 Associated Laboratory for Green Chemistry of the Network of Chemistry and Technology (LAQV-REQUIMTE), Department of Chemistry, University of Aveiro, 3810-193 Aveiro, Portugal; 2 Research Centre in Physical Activity, Health and Leisure (CIAFEL), Faculty of Sport, University of Porto, 4200-450 Porto, Portugal; 3 Experimental Pathology and Therapeutics Group, Research Center (CI-IPOP)/RISE@CI-IPOP (Health Research Network), Portuguese Oncology Institute of Porto (IPO-Porto)/Porto Comprehensive Cancer Center (P.CCC), 4200-072 Porto, Portugal; 4 Department of Medical Sciences, Institute of Biomedicine—iBiMED, University of Aveiro, 3810-193 Aveiro, Portugal; 5 Laboratory for Integrative and Translational Research in Population Health (ITR), 4050-600 Porto, Portugal

**Keywords:** cancer patients, chemotherapy, skeletal muscle, muscle wasting, molecular changes

## Abstract

Paraneoplastic conditions such as cancer cachexia are often exacerbated by chemotherapy, which affects the patient’s quality of life as well as the response to therapy. The aim of this narrative review was to overview the body-composition-related changes and molecular effects of different chemotherapy agents used in cancer treatment on skeletal-muscle remodeling. A literature search was performed using the Web of Science, Scopus, and Science Direct databases and a total of 77 papers was retrieved. In general, the literature survey showed that the molecular changes induced by chemotherapy in skeletal muscle have been studied mainly in animal models and mostly in non-tumor-bearing rodents, whereas clinical studies have essentially assessed changes in body composition by computerized tomography. Data from preclinical studies showed that chemotherapy modulates several molecular pathways in skeletal muscle, including the ubiquitin–proteasome pathway, autophagy, IGF-1/PI3K/Akt/mTOR, IL-6/JAK/STAT, and NF-κB pathway; however, the newest chemotherapy agents are underexplored. In conclusion, chemotherapy exacerbates skeletal-muscle wasting in cancer patients; however, the incomplete characterization of the chemotherapy-related molecular effects on skeletal muscle makes the development of new preventive anti-wasting strategies difficult. Therefore, further investigation on molecular mechanisms and clinical studies are necessary.

## 1. Introduction

According to the International Agency for Research on Cancer, there were 19.3 million new cancer cases worldwide in 2020 [1]. Cancer incidence is expected to increase by 56.7% over the next two decades, with 30.2 million cases estimated in 2040 [2]. Cancer has been associated with muscle wasting, which is often exacerbated by chemotherapy, one of the most common treatments [3]. Despite the plethora of benefits presented by chemotherapy regimens, there are still significant side effects to consider, which affect people differently. Nausea, loss of appetite, tiredness, fatigue, and weight loss are some of the side effects often experienced by patients undergoing chemotherapy treatments [4,5,6]. Some of these side effects are explained by the impact of chemotherapy on skeletal muscle (SkM) [7,8]. For instance, cancer patients receiving chemotherapy often experience body-weight loss, which can be attributed to SkM wasting. Such SkM loss may further exacerbate cancer-associated cachexia, a multifactorial syndrome accompanied by systemic inflammation, metabolic disarrangement, anorexia, and insulin resistance. This syndrome affects up to 80% of cancer patients [9,10].

Since SkM loss has a negative impact on the quality of life of cancer patients and is a marker of poor prognosis, the comprehension of the molecular mechanisms underlying chemotherapy-induced SkM wasting is of major importance to guide the development of anti-wasting strategies [11,12]. Therefore, this review overviews the literature regarding the effect of different chemotherapeutic agents on body-composition-related indexes and on SkM mass, function, and molecular changes.

## 2. Materials and Methods

A search on different databases such as Web of Science, Scopus, and Science Direct was performed and included a combination of “skeletal muscle” with other key associated concepts, including “chemotherapy”, “atrophy”, “mass loss”, “muscle wasting”, “muscle dysfunction”, and “cancer”. Only original articles on the effects of chemotherapeutic agents on SkM were considered. Data collected were organized according to the effects of cytotoxic agents on SkM in preclinical studies, in vitro/ex vivo (Table A1) and animal (Table 1) studies, and clinical (Table 2) studies. From the 77 selected papers, 2 were in vitro studies, 1 was ex vivo, 20 were performed with animal models, 53 were clinical studies, and 1 included in vitro and clinical data.

**Table 1:** Studies using animal models (mice and rats) to assess the effects of chemotherapy on SkM.

Chemotherapy	Animal Model/Muscle	Tumor Data	Main Results	Ref.
Doxorubicin				
15 mg/kg IP injection	Male, C57BL/6 mice, 7-week-old (*unspecified muscle*)	Non tumor bearing	↑atrogin-1, MuRF-1 mRNA	[13]
20 mg/kg IP injection	Adult male, NMRI mice, TNFR1 receptor-deficient *(EDL*)	Non tumor bearing	↓ body weight, EDL muscle weight (20%), estimated CSA, maximal absolute force (40 ± 2%), maximal specific force (28 ± 5%), specific (59 ± 5%)	[14]
20 mg/kg IP injection	Male, Sprague-Dawley rats, 8–10 weeks old (*gastrocnemius*)	Non tumor bearing	↓ body weight, whole-body fat, lean mass, maximal respiratory capacity (72 h after), energy expenditure ↑ mitochondrial H_2_O_2_ emission	[15]
1.5/4.5 mg/kg IP injection	Male, Sprague Dawley rats (*plantaris, soleus, gastrocnemius*)	Non tumor bearing	1.5 mg/kg ↑ [NO] intra in *soleus* at 24 h ↑ [NO] interstitial at 24, 48, 72, 96, and 120 h 4.5 mg/kg ↓ [NO]intra in *soleus* at 24 h and 48 h ↓ [NO]intra in white *gastrocnemius* at 4 h and 48 h ↑ [NO]interstitial at 96 h	[16]
15 mg/kg IP injection	Male, Wistar rats,~14 weeks old (*EDL*)	Non tumor bearing	↓ skeletal muscle, CSA, IRS-1, and GSK3-b mRNA, GLUT4, AMPK α (pT172) levels, activity of mitochondrial complex 3, IL-10 levels ↑ AST, uric acid, corticosterone/testosterone ratio, insulin, glucose, FFA, activity of mitochondrial complex 1 = IL-6 and TNF-α levels	[17]
12 mg/kg (3 injections of 4 mg/kg separated by two weeks) IP injection	Female, Sprague-Dawley rats, 8 weeks old ovariectomized (*soleus and EDL*)	Non tumor bearing	↓ pooled-fiber CSA in the *soleus*, fiber CSA in the EDL, Pax7-positive satellite cell in the *soleus* (38 ± 4%), capillary content in the *soleus* (35 ± 5%), MGF mRNA in the EDL ↑ MYF5 mRNA in the *soleus* = Ki67+ satellite cells, capillary content of the EDL	[18]
20 mg/kg IP injection	Male, C57BL/6NJ mice, 10–24 weeks old (*EDL*)	Non tumor bearing	↓ absolute respiratory sensitivity, membrane potential, specific force, fatigue resistance, rate of sarcoplasmic reticulum Ca^2+^ uptake ↑ soleus half-relaxation time	[19]
Cisplatin				
15 mg/kg (3 injections of 5 mg/kg) IV injection	Male, CD2F1 mice, 7–8 weeks old (*gastrocnemius*)	C26 adenocarcinoma cell-line injection	↓ MuRF-1 mRNA	[20]
12 mg/kg (4 daily injections of 3 mg/kg) IP injection	Male, C57BL/6J, mice 8–9 weeks old, 23–27 g (*quadriceps*)	Non tumor bearing	↓ body weight, muscle mass, food intake, myofiber diameters, p-Akt, p-FOXO3a, IGF mRNA ↑ FOXO3, FOXO3a, MAFbx, atrogin-1, MuRF-1, p21 and Mstn mRNA, p-Smad2 = FOXO1 mRNA	[21]
2.5 mg/kg (for up to 2 weeks) IP injection	Male, mice CD2F1, 8 weeks old (*quadriceps*)	Non tumor bearing	↓ body weight (−6.6 g), muscle strength (−23%) ↑atrogin-1 mRNA	[22]
Oxaliplatin				
20 mg/kg (4 injections of 5 mg/kg over 6 days) Or 30 mg/kg (12 injections of 2.5 mg/kg over 17 days)	Male, mice C57BL/6J, 8 weeks old (*gastrocnemius and soleus*)	Non tumor bearing	↓ body weight (20 mg/kg and 30 mg/kg), muscle weights and average speed (30 mg/kg) ↑ p-STAT3 (Tyr705) (20 mg/kg) in the *gastrocnemius*, atrogin-1, BNIP3, and FOXO3 mRNA (30 mg/kg) = FOXO1 mRNA (30 mg/kg)	[23]
Docetaxel				
20 mg/kg IV injection	Female, mice, 8–11 weeks old (*EDL, soleus, and gastrocnemius*)	Non tumor bearing	Repeated: ↓ body mass (1–3%), *soleus* muscle mass (9%), EDL muscle mass (7%), *gastrocnemius* muscle mass (10%), muscle cross-sectional area *soleus*, and EDL ↓ specific force of *soleus* muscles (17%) (acute)	[24]
Sorafenib				
90 mg/kg intragastrically	Male, Wistar rats, 5 weeks old (*gastrocnemius*)	Ascites hepatoma cells	↑ weight of *Soleus* (5 mg/100 g), of EDL (9 mg/100 g), of *gastrocnemius* (105 mg/100 g), grip strength	[25]
5-fluorouracil (5-FU)				
35 mg/kg IP injection	Male, C57BL/6 mice, 14 weeks old (*quadriceps and gastrocnemius*)	Non tumor bearing	↓ body weight (2–8%), average daily food intake (20.5% g/day), IL-1β (95%) and IFNγ (75%) mRNA =IL-6, TNF-α, and MCP-1 mRNA	[26]
23 mg/kg IP injection	Male, Balb/c mice, 6 weeks old (*soleus and EDL*)	Non tumor bearing	↓ desmin, dystrophin, p-Akt (Ser473), ↑ p-p38, p-65 subunit of NF-κB =Ankrd2, TFAM, PGC1α, PGC1β, DRP-1, and OPA-1 protein expression	[27]
Combinations of chemotherapy agents				
doxorubicin (10 mg/kg) and dexamethasone (2.5 mg/kg) (every 3 weeks for a maximum of 4 cycles on days 1–5 of each cycle) IP injection	Female, C57BL/6 mice, 9 weeks old (*lower limb*)	Non tumor bearing	↓ weight (23%), CS activity, Parkin levels, ratio Parkin/VDAC ↑ ROS production per unit respiration	[28]
gemcitabine (1000 mg/m^2^ per 3 days) cisplatin (75 mg/m^2^/week) (for 21 days) IP injection	Female, athymic nude mice (BALB/c), 7 weeks old, ~25 g *(gastrocnemius and soleus*)	T24 human bladder-cancer cell	↓ body weight (28.3 ± 1.8%) ↑ ActRIIB, FOXO3, MuRF 1, atrogin-1, myostatin, and activin A mRNA, muscular proteasome activity (chymotrypsin)	[29]
folfiri [5-fluorouracil (50 mg/kg), leucovorin (90 mg/kg), CPT-11 (24 mg/kg)] (twice a week for five consecutive weeks) IP injection	Male, CD2F1 mice, 8 weeks old *(quadriceps and gastrocnemius muscles)*	C26 adenocarcinoma cells	↓ body weight (15%), *gastrocnemius* mass (11%), *quadriceps* mass (20%), 15 calcium-binding proteins, MYOZ2 levels ↑ PSMA6, UBA1, KERA, LAMA2 protein levels	[30]
folfiri (5-fluorouracil, leucovorin, CPT-11) (for up to 5 consecutive weeks) IP injection	Male, CD2F1 mice (*tibialis anterior, EDL, gastrocnemius and quadriceps*)	Non tumor bearing	↓ body weight (10%), *quadriceps* muscle (23%), muscle force (17%), size of oxidative and glycolytic fibers, p-Akt, PGC1α, and PGC1β levels, cytochrome C, SDH activity, Ucp1, Cidea1, Acot2, and Fhl3 mRNA, *gastrocnemius* and *tibialis* anterior mass, fiber size and oxidative fibers in the *tibialis*, mitochondria density and size ↑ Alb, Fga, Fgb, Dnah5, Fabp1, Apoa1, Apob, Apoa2, Prkcz, and Scd2 mRNA, glycolytic fibers in the *tibialis*, p-ERK1/2, p-p38 MAPKs, ROS levels =Atrogin-1, MuRF-1, Fbxo21, Fbxo30, and Fbxo31 mRNA	[31]
folfox (5-fluorouracil, leucovorin, oxaliplatin) (for up to 5 consecutive weeks) IP injection	Male, CD2F1 mice (*tibialis anterior, EDL, gastrocnemius and quadriceps*)	Non tumor bearing	↓ muscle mass, PGC1α, PGC1β, cytochrome C, SDH activity, oxidative fibers in the *tibialis*, mitochondria density and size ↓ oxidative fibers and ↑ glycolytic fibers ↑ p-ERK1/2, p-p38 MAPKs, PGC1α, PGC1β, ROS levels = atrogin-1, MuRF-1, Fbxo21, Fbxo30, and Fbxo31 mRNA	[31]
folfiri [5-fluorouracil (50 mg/kg), leucovorin (90 mg/kg), and CPT-11 (24 mg/kg)] (twice a week) IP injection	Male, CD2F1 mice, 8 weeks old (*gastrocnemius*)	Non tumor bearing	↓ muscle weight, muscle-grip strength, fiber CSA (40–50%), PDH levels, ATP content, creatine phosphate ↑ AMP content, ROS levels = hexokinase activity, lactate, BCAA levels	[32]
	Male, CD2F1mice, 8 weeks old (*gastrocnemius*)	C26 adenocarcinoma cells	↑ muscle weight, ROS levels ↓ muscle-grip strength, fiber CSA (40–50%), PDH and hexokinase activity, ATP and creatine phosphate content, acetylcarnitine = lactate, BCAA levels	[32]

↑ increase; ↓ decrease; = no change. Abbreviations: Acot2: Acyl-CoA thioesterase 2; ActRIIB: activin receptor type-2B; Akt: protein kinase B; Alb: albumin; AMP: adenosine monophosphate; AMPk: 5’ AMP-activated protein kinase; Ankrd2: ankyrin repeat domain 2; Apoa: apolipoprotein A; Apob: apolipoprotein B; AST: aspartate aminotransferase; ATP: adenosine triphosphate; BCAA: branched-chain amino acids; Bnip3: BCL2 interacting protein 3; Cidea: death activator; CPT-11-irinotecan; CS: citrate-synthase activity; Dnah5: dynein axonemal heavy-chain 5; DRP-1: dynamin-related protein 1; ERK1/2: extracellular signal-regulated kinase 1/2; Fabp1: fatty-acid-binding protein 1; Fbxo21: F-box protein 21; Fbxo30: F-box protein 30; Fbxo31: F-box protein 31; FFA: free fatty acids; Fhl3: four and a half LIM domains 3; FOXO1: forkhead box O1; FOXO3: forkhead box O3; GLUT4: insulin-responsive glucose transporter; GSK3-b: glycogen-synthase kinase 3 beta; HIF-1α: hypoxia-inducible factor 1-alpha; IFNγ: cytokine type II interferon; IGF: insulin-like growth factors; IGF1R: insulin-like growth factor-1 receptor; IL-10: interleukin-10; IL-15: interleukin-15; IL-1β: interleukin 1 beta; IL-6: interleukin-6; IRS-1: insulin-receptor substrate 1; KERA and LAMA2: structural proteins; LDH: lactate dehydrogenase; MAFbx: F-box-only protein 32; MAPKs: mitogen-activated protein kinase; MEF2C: myocyte-enhancer factor 2C; MGF: mechano growth factor; Mstn: myostatin; MurRF-1: muscle RING-finger protein-1; Myf5: myogenic factor 5; MYOD1: myogenic differentiation 1; MYOZ2: myozenin 2; NFkB: nuclear factor kappa-light-chain enhancer of activated B cells; [NO]: concentration of nitric oxide; OPA-1: OPA1 mitochondrial dynamin-like GTPase; PDH: pyruvate dehydrogenase; PDK4: pyruvate dehydrogenase kinase 4; PGC-1α: peroxisome proliferator-activated receptor-gamma coactivator; PGC-1β: peroxisome proliferator-activated receptor-γ coactivator-1β; phosp.: phosphorylation; Scd2: stearoyl-CoA desaturase-2; SDH: succinate dehydrogenase; Smad2: SMAD family member 2; STAT3: signal transducers and activators of transcription 3; TFAM: transcription factor A mitochondrial; TNF: tumor-necrosis factor; TNFR1: tumor-necrosis-factor receptor 1; TNF-α: tumor-necrosis-factor alpha; UBA1 and PSMA6: proteasomal components; Ucp1: thermogenin; VDAC: voltage-dependent anion channels.

**Table 2:** Effects of chemotherapy in cancer patients.

Authors/Year	Chemotherapy	Cohort Characterization	Main Result
Poterucha et al., 2011 [33] USA	Bevacizumab 5 or 7.5 mg/kg plus fluoropyrimidine, oxaliplatin, and leucovorin or irinotecan-based regimen or single-agent capecitabine or 5-fluorouracil and leucovorin	57 patients with metastatic CRC (59 (26–84) yo, M: 53%); March of 2004 to 2007 (CT at L3 level)	↓ Muscle area (3 cm^2^)
Awad et al., 2012 [34] UK	Epirubicin/cisplatin/5-fluorouracil or cisplatin/5-fluorouracil (neoadjuvant)	47 patients with oesophagogastric cancer (63 ± 12 yo, M: 72.3%) (CT at L3 level)	↓ SMA (9.6 cm^2^)
Ida et al., 2014 [35] Japan	Docetaxel (60 mg/m^2^), 5-fluorouracil (350 mg/m^2^), and cisplatin (6 mg/m^2^) (neoadjuvant)	30 patients with EC (65 (53–75) yo, M: 83.3%) August 2010 to April 2013 (BIA)	= BMI, SkM
Cooper et al., 2014 [36] Hershey	Gemcitabine and cisplatin + 30 Gy x 10 concurrent gemcitabine (neoadjuvant chemoradiation; 12 weeks)	89 with resectabel PDAC patients (63 (38–79) yo, M: 55%) (CT at L3 level)	↓ SkM (1.2 cm^2^/m^2^)
Kimura et al., 2015 [37] Japan	Single-agent chemotherapy (docetaxel, gemcitabine, or vinorelbine), platinum-based chemotherapy (cisplatin + pemetrexed, carboplatin + paclitaxel ± bevacizumab, carboplatin + pemetrexed ± bevacizumab, cisplatin + docetaxel, carboplatin + S-1, nedaplatin + docetaxel, or carboplatin + gemcitabine), or molecular targeted treatment (gefitinib or elrotinib)	134 patients with NSCLC (66 (35–86) yo, M: 59.7%) January 2010 to August 2011 (CT at L3 level)	↓ LSMI (1.3 cm^2^/m^2^)
Yoon et al., 2015 [38] Japan	5-fluorouracil (1000 mg/m^2^), cisplatin (60 mg/m^2^), plus radiotherapy (40 to 50 Gy) (neoadjuvant)	248 patients with EC (63.5 ± 7.6 yo, M: 100%) 2005 to 2017 (CT at L3 level)	↓ BMI (0.8 kg), SMI (4.6 cm^2^/m^2^)
Reisinger et al., 2015 [39] Netherlands	Cisplatin/5-fluorouracil plus radiotherapy, paclitaxel/carboplatin radiotherapy, or epirubicin/cisplatin/capecitabine radiotherapy (neoadjuvant)	96 patients with EC (63 ± 9.2 yo, M: 83.3%) January 2008 to January 2012 (CT at L3 level)	↓ L3 index (2.5 cm^2^/m^2^)
Paireder et al., 2016 [40] Austria	Taxane-based, platinum-based combination of taxane/platinum, other/unknown, or chemoradiotherapy (neoadjuvant)	130 patients with EC (61.4 (30.8–81.0) yo, M: 81.5%) 2006 to 2013 (CT at L3 level)	↓ Male SMI (2.3 cm^2^/m^2^) ↓ Female SMI (2.8 cm^2^/m^2^) not significative
Rollins et al., 2016 [41] UK	Gemcitabine-based chemotherapy (palliative)	98 patients with pancreatic cancer or distal cholangiocarcinoma (64.8 ± 8.7 yo, M: 56.1%) 2006 to 2013 (CT at L3 level)	↓ SMA (7.4 cm^2^), SMI (2.6 cm^2^/m^2^), SMD (1.0 HU)
Heus et al., 2016 [42] Netherlands	Capecitabine 1500 mg + 28 × 1.8 Gy (total of 50.4 Gy) (neoadjuvant chemoradiation; 5 weeks)	74 patients with locally advanced RC (64.0 ± 10 yo, M: 53%) 2006 to 2013 (CT at L3 level)	↑ SMA (2.2 cm^2^)
Liu et al., 2016 [43] Japan	5-fluorouracil 800 mg/m^2^ and cisplatin or nedaplatin 80 mg/m^2^ +40 Gy × 20 (neoadjuvant chemoradiation)	84 patients EC (63 (40–74) yo, M: 85.7%) September 2008 to January 2015 (CT at L3 level)	↓ PMI (0.14 cm^2^/m^2^ in males and 0.32 cm^2^/m^2^ in females)
Mayanagi et al., 2017 [44] Japan	Platinum plus fluorouracil-based (neoadjuvant)	66 patients with thoracic EC (63.3 ± 8.0 yo; M: 86%) May 2004 to December 2013 (CT at L3 level)	↑ SkM (0.3 ± 3.0 cm^2^/m^2^)
Kakinuma et al., 2017 [45] Japan	Carboplatin + emetrexed + bevacizumab or cisplatin+ emetrexed + bevacizumab or carboplatin + gemcitabine or cisplatin + gemcitabine or carboplatin + paclitaxel or carboplatin + nab- paclitaxel or cisplatin + docetaxel or emetrexed	65 patients with NSCLC (67.2 ± 7.7 yo; M: 70.5%) January 2012 to December 2014 (CT at L3 level)	↓ SMI (4.4 cm^2^/m^2^), ΔSMA (11.5 cm^2^)
Levolger et al., 2017 [46] Netherlands	Capecitabine (825 mg/m^2^) and radiotherapy (50 Gy) (neoadjuvant)	122 patients with RC (61.0 (53.0–66.3) yo, M: 58.2%) August 2004 to December 2012 (CT at L3 level)	= SMI
Miyata et al., 2017 [47] Japan	5-fluorouracil (700 mg/m^2^), cisplatin (70 mg/m^2^), and adriamycin (70 mg/m^2^) or 5-fluorouracil (700 mg/m^2^), cisplatin (70 mg/m^2^), and docetaxel (70 mg/m^2^) (neoadjuvant)	94 patients with EC (64.2 ± 8.8 yo, M: 80.9%) January 2013 to August 2016 (BIA)	↓ SMM (0.1 kg) not significative ↑ Sarcopenia (47% to 53%)
Guinan et al., 2017 [48] Ireland	Etoposide, cisplatin, 5-fluorouracil or capecitabine or chemoradiotherapy (cisplatin/5-fluorouracil, 40 Gy or carboplatin/paclitaxel, 41.4 Gy) (neoadjuvant)	28 patients with EC (62.8 ± 8.2 yo, M: 82%) January 2014 to October 2016 (CT at L3 level)	↓ SMI (5.6 cm^2^/m^2^), overall HGS (4.3kg)
Naito et al., 2017 [49] Japan	Single-agent chemotherapy (docetaxel (60 mg/m^2^) or vinorelbine (25 mg/m^2^)) or platinum-based chemotherapy (carboplatin + paclitaxel (200 mg/m^2^) or cisplatin (75 mg/m^2^) + pemetrexed (500 mg/m^2^) or cisplatin (80 mg/m^2^) + gemcitabine (1000 mg/m^2^) or cisplatin (80 mg/m^2^) + vinorelbine (25 mg/)) or gefitinib (250 mg)	30 patients with NSCLC (74 (70–82) yo, M: 63.3%) January 2013 to January 2014 (CT at L3 level)	↓ L3 index (1.8 cm^2^/m^2^), HGS (0.7 kg)
Cho et al., 2017 [50] Korea	Gemcitabine/platinum or 5-fluorouracil/platinum chemotherapy (palliative)	524 patients with biliary tract cancer (61 ± 9.4 yo, M: 65,6%) 2003 to 2013 (CT at L3 level)	↓ SMI (5.35 cm^2^/m^2^), male SMI (5.72 cm^2^/m^2^), female SMI (4.61 cm^2^/m^2^)
Motoori et al., 2018 [51] Japan	Cisplatin (70 mg/m^2^), docetaxel (70 mg/m^2^), and 5-fluorouracil (700 mg/m^2^) (neoadjuvant)	83 patients with EC (65 (45–81) yo, M: 79.5%) January 2013 to December 2015 (BIA)	15 patients (18%) lost more than 5% of SkM
Lee et al., 2018 [52] Taiwan	Cisplatin (40 mg/m^2^) plus radiotherapy (neoadjuvant)	245 patients with cervical cancer (63.0 ± 12.7 yo, M: 0%) March 2004 to December 2015 (CT at L3 level)	↓ SMD (1.2 HU) ↓ SMI (0.3 cm^2^/m^2^) not significative
Panje et al., 2018 [53] Taiwan	Induction chemotherapy (docetaxel (75 mg/m^2^), cisplatin (75 mg/m^2^)) and radiochemotherapy (45 Gy; docetaxel (20 mg/m^2^) and cisplatin (25 mg/m^2^) with or without cetuximab (250 mg/m^2^)) (neoadjuvant)	300 patients with EC (61 (36–75) yo, M: 87.7%) May 2010 to December 2013 (CT at L3 level)	↓ L3 index (15.7 cm^2^/m^2^), SMA (14.6 cm^2^)
Järvinen et al., 2018 [54] Finland	Epirubicin–oxaliplatin–capecitabine or platin- and 5-fluorouracil-based therapy plus 45 Gy total dose of radiotherapy (neoadjuvant)	115 patients with EC (63 ± 9 yo, M: 74.8%) 2010 to 2014 (CT at L3 level)	50% of patients had severe SkM loss
Sandini et al., 2018 [55] Boston	FOLFIRINOX PAXG PEXG (neoadjuvant)	193 patients with borderline resectable and locally advanced PC (64 ± 11 yo, M: 50.3%) January 2013 to December 2015 (CT at L3 level)	↑ Lean mass, muscle gain (6.8 cm^2^) [FOLFIRINOX] SkM↓ 2.5 cm^2^ (no resection) ↑ 7.2 cm^2^ (resection)
Kays et al., 2018 [56] USA	FOLFIRINOX	53 patients with locally advanced and metastatic PDAC (59.5 ± 9.9 yo, M: 62.3%) July 2010 to August 2015 (CT at L3 level)	↓ SkM (3.5 kg), SMI (7.2%), SkM (0.7 HU)
Park et al., 2018 [57] Korea	Combined capecitabine and oxaliplatin (adjuvant 8 weeks)	136 patients with GC (55.0 (20–76) yo, M: 70.6%) May 2006 to April 2009 (CT at L3 level)	SMI: 48.3 cm^2^/m^2^ surgery only SMI: 44.8 cm^2^/m^2^ adjuvant chemotherapy
Guigni et al., 2018 [58] Vermont	Cyclophosphamide or doxorubicin or trastuzumab (adjuvant)	13 patients with BC (66 ± 5 yo, M: 0%) *vastus lateralis*	↓ Single-muscle fiber CSA, MHC II fiber CSA, intermyofibrillar mitochondrial fractional area, average mitochondrial area, fractional content of MHC I proteins ↑ Oxidized Prx 3 = Myosin or actin protein, number of mitochondria
Okuno et al., 2018 [59] Houston	Oxaliplatin based, irinotecan based, bevacizumab, cetuximab/panitumumab (neoadjuvant)	169 patients with CRLM (56.2 ± 11.7 yo, M: 57.4%) January 2009 to December 2013 (CT at L3 level)	↓ SMI (0.52 cm^2^/m^2^) major mass loss (≥7%) was observed in all regiments
Lyon et al., 2019 [60] Rochester	Gemcitabine, cisplatin (neoadjuvant)	183 patients with MIBC (65 (57–72) yo, M: 85%) 2000 to 2016 (CT at L3 level)	↓ SMA (3.6 cm^2^) ↓ SMI (1.1 cm^2^)
	Methotrexate, vinblastine, adriamycin , and cisplatin or cisplatin, methotrexate, and vinblastine (neoadjuvant)		↓ SMA (7.0 cm^2^) ↓ SMI (2.3 cm^2^)
	Other cisplatin-based (neoadjuvant)		↓ SMA (3.9 cm^2^) ↓ SMI (1.2 cm^2^)
Degens et al., 2019 [61] Netherlands	Paclitaxel, carboplatin, bevacizumab, and nitroglycerin	111 patients with NSCLC (61 (39–79) yo; M: 54%) (CT at L3 level)	↓ SkM CSA (1.2 cm^2^/m^2^) ↓SkM CSA (2.7% cm^2^/m^2^)
Griffin et al., 2019 [62] Ireland	FOLFIRINOX; gemcitabine and nab-paclitaxel; gemcitabine; gemcitabine and oxaliplatin; gemcitabine and cisplatin/carboplatin; 5-fluorouracil (neoadjuvant)	78 patients with PC (64.2 ± 7.9 yo, M: 47%) 2012 to 2015 (CT at L3 level)	↓ SkM (8.4 cm^2^), LSMI (3.3 cm^2^/m^2^), MA (0.2 HU), estimated SMM (1.47 kg)
Matsuura et al., 2019 [63] Japan	S-1 (prodrug of 5-fluorouracil) 80 mg/m^2^ + cisplatin 60 mg/m^2^ or + cisplatin 60 mg/m^2^ + docetaxel 40 mg/m^2^ or + oxaliplatin 100 mg/m^2^ (neoadjuvant)	41 patients with advanced GC (72 (48–82) yo, M: 68.3%) January 2013 to December 2016 (CT at psoas muscle level)	↓ PMI (5.93%)
Rier et al., 2019 [64] Netherlands	FAC (5-fuourouracil 500 mg/m^2^, doxorubicin 50 mg/m^2^ and cyclophosphamide 500 mg/m^2^) or paclitaxel 80 mg/m^2^ (palliative)	98 patients with metastatic BC (FAC: 57.0 (49.5–67.0) yo, M: 0%) (Paclitaxel: 56.0 (48.0–62.5) yo, M: 0%) January 2000 to March 2016 (CT at L3 level)	FAC: ↓ MA (0.7 HU), LSMI (0.5 cm^2^/m^2^) Paclitaxel: ↓ MA (0.7 HU), ↑ LSMI (0.3 cm^2^/m^2^)
Fukuoka et al., 2019 [65] Japan	Capecitabine + oxaliplatin (CapeOx), capecitabine + oxaliplatin + cetuximab (CapeOx + cetuximab), modified FOLFOX6 or chemoradiotherapy (1.8 or 2.0 Gy and capecitabine (825–900 mg/m^2^) (neoadjuvant)	47 patients with RC (66 (27–88) yo, M: 74.5%) January 2010 to December 2016 (CT at navel level)	↓ PMI (12.4 cm^2^/m^2^; 4.3%)
Nardi et al., 2019 [66] Italy	Oxaliplatin 100 mg/m^2^ plus 5-fluorouracil 200 mg/m^2^ plus a total of 41.4 Gy chemoradiotherapy (neoadjuvant)	52 patients with RC (63 (32–79) yo, M: 65%) January 2010 to March 2014 (CT at L3 level)	36.5% had SkM loss >2% 30.7% had SkM loss >5% ↓ SMA (0.48 cm^2^) not significative
Ozawa et al., 2019 [67] Japan	CDDP (80 mg/m^2^) and 5-fluorouracil (800 mg/m^2^/day) or chemoradiotherapy (CDDP (70 mg/m^2^), 5-fluorouracil (700 mg/m^2^/day and 30 Gy long-T) (neoadjuvant)	82 patients with EC (63.5 ± 7.5 yo, M: 86.6%) January 2008 to December 2013 (CT at L3 level)	↓ PMI (0.2 cm^2^/m^2^)
Ishida et al., 2019 [68] Japan	Docetaxel (70 mg/m^2^), cisplatin (70 mg/m^2^), and 5-fluorouracil (700 mg/m^2^) or adriamycin (35 mg/m^2^), cisplatin, (70 mg/m^2^), and 5-fluorouracil (700 mg/m^2^) (neoadjuvant)	165 patients with EC (Low PMI: 68.3 ± 6.4 yo, M:93%) (High PMI: 65.1 ± 9.9 yo, M: 85.3%) January 2010 to December 2013 (CT at L3 level)	↓ PMI (0.2 cm^2^/m^2^)
Huang et al., 2019 [69] China	Radical radiotherapy ± target therapy or concurrent chemoradiotherapy ± target therapy/adjuvant chemotherapy or induction chemotherapy + concurrent chemoradiotherapy ± target therapy/adjuvant chemotherapy or induction chemotherapy + radical radiotherapy	394 patients with nasopharyngeal cancer (46 (18–79) yo, M: 75.6%) January 2015 to December 2017 (CT at L3 level)	↓ SMA (13.1 cm^2^), SMI (4.7 cm^2^/m^2^)
Li et al., 2019 [70] China	5-fluorouracil (400 mg/m^2^), leucovorin (200 mg/m^2^), and radiotherapy (45 Gy to 50.5 Gy) (adjuvant)	153 patients with GC (52.1 (26–89) yo, M: 66.0%) January 2008 to December 2016 (CT at L3 level)	↓ SMI (1.6 cm^2^/m^2^)
Lee et al., 2019 [71] Taiwan	Paclitaxel 175 mg/m^2^ and carboplatin AUC5 and radiotherapy (neoadjuvant)	131 patients with endometrial cancer (54.3 ± 9.6 yo, M: 0%) 2008 to December 2016 (CT at L3 level)	↓ SMI (0.1 cm^2^/m^2^), SMD (1.0 HU), SMG (SkM gauge) (37.2) not significative
Yassaie et al., 2019 [72] New Zealand	Epirubcin/cisplatin/capecitabine chemotherapy (MAGIC protocol) or carboplatin/paclitaxel or chemoradiotherapy (CROSS) protocol (neoadjuvant)	53 patients with EC (loss of TPA ≤ 4%: 62.6 ± 6.7 yo, M: 85%) (loss of TPA >4%: 65.8 ± 8.0 yo, M: 97%) August 2008 to February 2018 (CT at L4 level)	↓ Total psoas area (TPA) (7.3%)
Kawakita et al., 2020 [73] Japan	Chemoradiotherapy (40–41.4 Gy and 5-fluorouracil (800 mg/m^2^/day) plus cisplatin or nedaplatin (80 mg/m^2^/day)) (neoadjuvant)	113 patients with EC (loss of PMI ≥ 20%: 65 (56–68) yo, M: 85.2%) (loss of PMI < 20%: 64 (59–68) yo, M: 85.9%) April 2009 to March 2017 (CT at L3 level)	↓ PMI (5.3%)
den Boer et al., 2020 [74] UK	Cisplatin–capecitabine, epirubicin–cisplatin–capecitabine, or other (neoadjuvant)	199 patients with gastro-oesophageal cancer (66.1 (28.4–80.0) yo, M: 79.4%) March 2016 to June 2019 (CT at L3 level)	↓ SMA (7.81 cm^2^), SMI (2.68 cm^2^/m^2^)
Grün et al., 2020 [75] Japan	FLOT protocol or CROSS protocol (neoadjuvant)	52 patients with EC (67.4 ± 12.0 yo, M: 86.5%) January 2018 to July 2019 (CT at L3 level)	↓ SMI (5.6 cm^2^/m^2^)
Ishibashi et al., 2020 [76] Japan	Cisplatin (80 mg/m^2^) and 5-fluorouracil (800 mg/m^2^) (neoadjuvant)	85 patients with EC (68.6 ± 0.9 yo, M: 89.0%) January 2009 to December 2014 (CT at L3 level)	↓ PMI (0.26 cm^2^/m^2^)
Kamitani et al., 2019 [77] Japan	DCF (docetaxel 70 mg/m^2^, cisplatin 70 mg/m^2^, 5-fluorouracil 700 mg/m^2^); divided DCF (docetaxel 35 mg/m^2^, cisplatin 6 mg/m^2^, 5-fluorouracil 350 mg/m^2^); FP (cisplatin 5 mg/m^2^, 5-fluorouracil 250 mg/m^2^) (neoadjuvant)	119 patients with EC (42 patients ≥ 66 yo and 48 patients (<66 yo, M: 86%) February 2007 to August 2018 (CT at L3 level)	↓ SMI (68.9%)
Fujihata et al., 2020 [78] Japan	FP therapy (5-fluorouracil and cisplatin) DCF therapy (docetaxel, cisplatin and 5-fluorouracil) (neoadjuvant)	99 patients with EC (68.0 (61.0–71.5) yo, M: 90%) August 2008 to June 2019 (CT at L3 level)	↓ SMI (1.87% cm^2^/m^2^), psoas major (0.4%), side abdominal muscles (3.0%), rectus abdominis (2.7%)
Dolly et al., 2020 [79] France	Bevacizumab 5 mg/kg body in 5-fluorouracil based regimens 7.5 mg/kg in capecitabine-based regimens	72 patients with metastatic CRC (64.2 ± 10.5 yo, M: 62.5%) January 2007 to December 2012 (CT at L3 level)	↓ SMM (8.1%)
Yoshino et al., 2020 [80] Japan	Paclitaxel and carboplatin or docetaxel and carboplatin or irinotecan and carboplatin (neoadjuvant)	75 patients with EOC (stage III/IV) (63.5 (43–81) yo, M: 0%) January 2010 to December 2017	↓ SMA (3.8 cm^2^)
Park et al., 2020 [81] South Korea	S-1/cisplatin or capecitabine and cisplatin, FOLFOX or XELOX (capecitabine and oxaliplatin), trastuzumab plus capecitabine and cisplatin, S-1 or capecitabine (palliative)	194 patients with advanced GC (65 (31–87) yo, M: 72.1%) September 2010 to December 2019 (CT at L3 level)	↓ SMI (4.5 cm^2^/m^2^; 11.3%) Male: ↓ SMI (4.3 cm^2^/m^2^; 10.1%) Female: ↓ SMI (4.5 cm^2^/m^2^; 12.8%)
Huang et al., 2020 [82] Taiwan	Platinum-based (adjuvant)	139 patients with EOC (stage III) (54.4 ± 10.3 yo, M: 0%) 2008 to 2017 (CT at L3 to the iliac crest level)	↓ SMI (0.8 cm^2^/m^2^), SMD (0.8 HU)
Kita et al., 2021 [83] Japan	-Cisplatin 70 mg/m^2^ -Adriamycin 35 mg/m^2^ -5-fluorouracil 700 mg/m^2^ (neoadjuvant)	87 patients with EC (stage IIA, IIB, III or IV) (EN group: 62.5 ± 8.1 yo, M: 72.3%) (PN group: 63.2 ± 7.4 yo, M: 82.5%) (CT at L3 level)	↓ SkM (2.1 cm^2^/m^2^)
Nakayama et al., 2021 [84] Japan	5-fluorouracil (800 mg/m^2^) and cisplatin (80 mg/m^2^), docetaxel (70 mg/m^2^), cisplatin (70 mg/m^2^), and 5-fluorouracil (700 mg/m^2^), 5-fluorouracil (800 mg/m^2^), cisplatin (80 mg/m^2^), and radiotherapy (40 Gy) (neoadjuvant)	63 patients with EC (66.3 ± 8.0 yo, M: 90.5%) January 2008 to December 2015 (CT at L3 level)	↑ Male PMI (0.05 cm^2^/m^2^), female PMI (0.5 cm^2^/m^2^) not significative
Zhang et al., 2021 [85] China	SOX, XELOX/FOLFOX, or chemoradiotherapy (neoadjuvant)	110 patients with GC (61.5 (53–67) yo, M: 72.7%) January 2016 to December 2018 (CT at L3 level)	= Total SkM
Rinninella et al., 2021 [86] Italy	FLOT docetaxel at 50 mg/mq, oxaliplatin at 85 mg/mq, leucovorin at 200 mg/mq, and 5-fluorouracil 2600 mg/mq (neoadjuvant)	26 patients with advanced GC (63.3 ± 11.2 yo, M: 69.2%) April 2019 to January 2020 (CT at L3 level)	↓ Weight (5 kg), BMI (1.8 kg/m^2^), SMA (6.5 cm^2^), SMI (2.22 cm^2^/m^2^)

↑ increase; ↓ decrease; = no change. Ages are presented as mean ± standard deviation or mean with the range of the minimum and maximum age. Abbreviations: BIA: multifrequency bioelectrical impedance CRLM: colorectal liver-metastases cancer; CSA: cross-sectional area; CT: computerized tomography; EC: esophageal cancer; EN: enteral nutrition; EOC: advanced epithelial ovarian cancer; EOC: advanced-stage epithelial ovarian cancer; FOLFIRINOX: fluorouracil, irinotecan, oxaliplatin, leucovorin, and folic acid: GC: gastric cancer; HGS: hand-grip strength; HU: Hounsfield units; LSMI: lumbar skeletal-muscle index; M: male; MA: muscle attenuation; mBC: metastatic breast cancer; mCRC: metastatic colorectal cancer; MHC: myosin heavy chain; MIBC: muscle-invasive bladder cancer; NSCLC: non-small-cell lung cancer; PAXG: cisplatin, capecitabine, gemcitabine, and nanoparticle albumin-bound paclitaxel; PDAC: pancreatic ductal adenocarcinoma; PEXG: cisplatin, epirubicin, capecitabine, and gemcitabine; PMI: psoas-muscle index; PN: parental nutrition; RC: rectal cancer; SkM: skeletal muscle; SMA: skeletal-muscle area; SMD: skeletal-muscle radiodensity; SMI: skeletal-muscle index; SMM: skeletal-muscle mass; TPA: total *psoas* area; Yo: years.

## 3. Overview of Selected Studies

In vitro studies (Table A1) [58,87,88,89] on the effect of chemotherapy in cell lines or in ex vivo muscle tested different chemotherapeutic agents and doses, making it difficult to establish a response pattern to chemotherapy. Regarding preclinical studies [13,14,15,16,17,18,19,20,21,22,23,24,25,26,27,28,29,30,31,32], the majority used male mice. From the 20 articles listed in Table 1, one [14] reported the effects of chemotherapy on knockout mice for the tumor-necrosis-factor receptor 1 (TNFR1) and only five focused on the effects of chemotherapy in animals with cancer (commonly C26 adenocarcinoma-cell inoculation). Indeed, most of the studies (n = 20) herein reviewed evaluated the effects of chemotherapy in healthy animals, making it difficult to translate the molecular findings to the clinical-oncology setting. The most studied chemotherapeutic agent was doxorubicin (7 papers), a potent anticancer drug known for its dose-dependent toxicity in many organs, including the heart and SkM [90]. The most studied SkM was the *extensor digitorum longus* (EDL) muscle, a fast-twitch muscle; however, other muscles, such as the slow-twitch *soleus*, the mixed-muscle *gastrocnemius*, and the fast-twitch *quadriceps* were also analyzed. Differences in both morphological and molecular changes induced by chemotherapy reported among studies (Table 1) can be related to the different chemotherapeutic agent, dosages, animal model and age, and SkM analyzed.

Among clinical studies, 38 focused on the effect of neoadjuvant chemotherapy or chemoradiation, 4 on adjuvant therapy, 4 involved palliative care, and 8 studies did not mention the moment of chemotherapy. Esophageal cancer was the most common cancer studied, followed by colorectal and gastric, pancreatic and lung, ovarian, breast and esophagogastric, and lastly, nasopharyngeal, cervical, endometrial, muscle-invasive bladder, and biliary-tract cancer. Differences between and within studies were found for other factors such as stage of cancer, number of chemotherapy cycles, duration of treatment, and type of treatment, as observed in Table 2, which made data interpretation and integration difficult.

### 3.1. Effects of Chemotherapy on Skeletal-Muscle Mass and Function

Loss of muscle mass and function negatively impacts the quality of life of cancer patients and is a marker of poor prognosis and survival [11,12,91]. In addition, SkM loss is also associated with reduced tolerance to anticancer treatments and exercise intolerance (fatigue) [11,12,91]. In most clinical studies (Table 2 [33,34,35,36,37,38,39,40,41,42,43,44,45,46,47,48,49,50,51,52,53,54,55,56,57,59,60,61,62,63,64,65,66,67,68,69,70,71,72,73,74,75,76,77,78,79,80,81,82,83,84,85,86]), SkM measurements rely on multifrequency bioelectrical impedance and mostly on computerized tomography (CT), a technique widely used for clinical purposes because it allows for the visualization of muscle cross-sections [92]. These studies showed that SkM index (SMI) and psoas muscle index (PMI) decreased in patients submitted to chemotherapy, independently of the drugs used. However, the SkM area (SMA) was reported to increase in one study published by Heus et al. [42]. This result was unexpected; however, chemoradiation was used in this study. Although the muscles measured were not in the direct field of radiation, chemoradiation may have decreased the inflammatory state, preventing SkM mass loss [42]. In addition, the lumbar SkM index (LSMI) seems to vary with the chemotherapeutic regimen used; it seems to be higher when patients are treated with paclitaxel [64] and lower in patients treated with different chemotherapy drugs [37,62,64]. All these body-composition-related indexes, such as SMI, can be useful as prognostic factors for overall survival [93]. Nonetheless, the application of CT scans and other imaging approaches in the analysis of body composition of cancer patients for the evaluation of SkM wasting is unrealistic, not only due to difficulty of data interpretation in a busy clinical setting but also due to its high costs. In clinical studies, SkM function has been assessed by hand-grip strength, which was diminished in patients with esophageal cancer and non-small-cell lung cancer treated with multiple chemotherapy therapies [48,49]. Additionally, a cross-sectional area of *vastus lateralis* was reported to be diminished with a lower cross-sectional area of type II fibers in breast-cancer patients [58].

In preclinical studies, EDL, *soleus*, *gastrocnemius*, and *quadriceps* muscles were analyzed, and loss of mass was reported for all these muscles following chemotherapy, which resulted, in most of the studies, in a lower body weight, and in some cases, diminished food intake was reported [21,26]. However, when sorafenib [25] and FOLFIRI [32] were administered, the mass of *gastrocnemius* and *tibialis anterior* increased [25,32]. The specific force of *soleus* [24] and maximal specific force of EDL were decreased [14], as well as average speed (which was measured using a software that processed beam breaks quantified by using locomotion and rearing records) [23]. Diminished SkM function assessed by the forelimb-grip strength was reported in healthy and tumor-bearing animals treated with FOLFIRI [32]. SkM-phenotype remodeling was reported since the ratio of glycolytic to oxidative fibers in *quadriceps* muscles increased [31], suggesting an enhanced susceptibility to fatigue following treatment with FOLFIRI or FOLFOX, and a decrease in *quadriceps* myofiber diameter after cisplatin treatment was also stated [21]. Glycolytic fibers are particularly susceptible to cancer-induced atrophy, as observed in muscle biopsies from human cancer patients [94]. Consequently, it is expected that chemotherapy will worsen the atrophy of type II fibers in these patients. Nevertheless, to the best of our knowledge, no preclinical studies have examined the effects of chemotherapy on cancer-induced muscle-fiber atrophy.

### 3.2. Molecular Impact of Chemotherapy on Skeletal Muscle

During and following chemotherapy, catabolic pathways seem to overcome the anabolic ones, potentiating the muscle wasting often observed in cancer patients. The following subsections overview the signaling pathways modulated in SkM by different anticancer agents from an integrated perspective. In overall, SkM mass is regulated by distinct pathways, namely, the catabolic ubiquitin–proteasome pathway (UPP), the autophagy–lysosome pathway (ALP), the myostatin/high-affinity type-2 activin receptor (ActRIIB) pathway, and the anabolic insulin-like growth factor 1 (IGF-1)/phosphatidylinositol-3-kinase (PI3K)/Akt (or protein kinase B, PKB)/mammalian target of rapamycin (mTOR) pathway [10,95]. Both muscle wasting and growth result from the balance between these pathways in favor of catabolic or anabolic processes, respectively [10,95,96,97]. Other signaling pathways contribute to chemotherapy-induced SkM remodeling like metabolism reprogramming, satellite-cell activation, and inflammation-associated pathways, such as interleukin 6 (IL-6)/Janus kinase-signal transducer and the activator of transcription protein (JAK/STAT) and nuclear-factor kappa-light-chain enhancer of activated B cells (NF-κB).

#### 3.2.1. Metabolic Reprogramming

Following chemotherapy, the metabolism becomes less reliant on mitochondria for energy generation [98], reflected in the decreased density (given the citrate synthase (CS) activity) and functionality of this organelle and a more glycolytic phenotype. This metabolic switching was reported in *quadriceps* muscle from male mice treated with FOLFIRI and FOLFOX [31]. In *vastus lateralis* of breast-cancer patients, a lower average of mitochondrial area, particularly of the intermyofibrillar mitochondria subpopulation, was reported; however, the number of mitochondria evaluated by fluorometric dyes was not affected by chemotherapy [58]. Mitochondrial density reflects the balance between biogenesis and clearance by mitophagy. The expression of peroxisome proliferator-activated receptor-gamma coactivator 1 alpha (PGC1α), a master player of mitochondrial biogenesis, was negatively impacted in the *quadriceps* of male mice following FOLFIRI and FOLFOX [31]. Still, unchanged levels of this coactivator were seen in *soleus* and EDL muscles from male mice treated with 5-fluorouracil (5-FU) [27]. PGC1α transcriptionally regulates the mitochondrial transcription factor A (TFAM), which is necessary for mtDNA maintenance [99]. Yet, TFAM levels were also found to be unchanged in *soleus* and EDL muscles [27]. Regarding mitophagy, decreased levels of parkin, an E3 ligase that synergistically acts with PTEN-induced kinase 1 (PINK1) for mitochondria engulfment by autophagosomes, was reported in lower-limb muscles treated with a combination of doxorubicin and dexamethasone [28]. Nonetheless, no changes in the levels of the mitochondria-fusion protein OPA-1 and -fission protein DRP-1 were observed [27]. Down-regulation of mitophagy may lead to the accumulation of dysfunctional mitochondria once no changes in biogenesis seem to occur and the density of this organelle is maintained. In fact, a significant decrease in ATP production was reported in the *tibialis anterior* of both non-tumor-bearing and tumor-bearing male mice treated with FOLFIRI [32]. Nevertheless, when the activity of mitochondrial oxidative phosphorylation (OXPHOS) complexes was measured in EDL of male rats, complex I activity was increased whereas complex III was decreased following treatment with doxorubicin [17]. Succinate dehydrogenase (SDH) activity and cytochrome c content were also diminished [31], suggesting a decreased oxidative capacity of SkM.

SkM is highly reliant on free fatty acids for energetic purposes. Fatty acids are oxidized in mitochondria and, thus, diminished fatty-acid oxidation (FAO) can be expected given the lower content of functional mitochondria in treated SkM. On the other hand, some proteins related to fatty-acid metabolism were reported to be upregulated by chemotherapy. The content of fatty-acid-binding protein (FABP) 1 increased in the *quadriceps* of male mice treated with FOLFIRI [31]. This protein belongs to the FABPs family, whose main function is to facilitate the transport of long-chain free fatty acids into the cell [100]. Moreover, the expression of stearoyl-CoA desaturase-2 (SCD2), one of the enzymes responsible for monounsaturated-fatty-acid synthesis [101], was also found to be increased in the *quadriceps* [31], suggesting increased fatty-acid production in wasted muscle. Additionally, the expression of acyl-CoA thioesterase (Acot) 2, which hydrolyzes coenzyme A (CoA) esters into free fatty acid and CoA, was diminished in the *quadriceps* after FOLFIRI treatment [31]. If not balanced by FAO, increased accumulation of free fatty acids and, eventually, of triglycerides can be presumed in wasted SkM. In fact, impaired FAO due to altered mitochondrial function was already associated with the accumulation of intramyocellular lipid droplets in muscle fibers of cachectic-cancer patients [102]. Other proteins associated with lipid metabolism were reported to be modulated by chemotherapy, including the expression of the apolipoproteins A and B (Apoa1, Apoa2, and Apob), essential in cholesterol metabolism, which were observed to be upregulated in the *quadriceps* muscles [31].

Amino acids derived from SkM proteolysis can also support energy generation [103]. Enhanced oxidation of branched-chain amino acids (BCAA; meaning Leu, Ile, and Val) in SkM was associated with the development of muscle wasting in cachectic subjects [104]. Still, BCAAs play a role in SkM that goes beyond energetic metabolism. BCAAs, particularly Leu, may activate mTORC1 signaling, boosting protein synthesis in SkM [105]. However, no changes in the levels of BCCAs induced by FOLFIRI were observed [32].

The accumulation of dysfunctional mitochondrial makes SkM more reliant on glucose oxidation for ATP generation. Thus, the glucose-uptake and -glycolysis rate should increase. Insulin regulates glucose uptake in SkM by promoting, among other cellular processes, glucose transporter type 4 (GLUT4) translocation from intracellular vesicles to the sarcolemma. However, GLUT4 content was found to be decreased in male rats’ EDL when treated with doxorubicin [17], which may indicate impaired insulin signaling, as supported by the observed diminished content of IGF-1 tyrosine-receptor insulin-receptor substrate 1 (IRS-1) [17]. In this same study, the levels of phosphorylated 5′ AMP-activated protein kinase (AMPK), another regulator of GLUT4 translocation, were found to be down-regulated [17]. Still, the expression of mitogen-activated protein kinases (MAPKs), such as extracellular signal-regulated kinase 1/2 (ERK1/2), known to be activated by insulin, increased in male mice’s *quadriceps* after FOLFIRI and FOLFOX [31]. Moreover, the levels of lactate were unchanged, whereas the activity of pyruvate dehydrogenase (PDH) complex, which converts pyruvate into acetyl-CoA, decreased in the *tibialis anterior* from healthy and tumor-bearing male mice treated with FOLFIRI [32]. The activity of the glycolytic enzyme hexokinase was unchanged in the *tibialis anterior* from healthy mice but decreased in the same muscle from mice with C26 adenocarcinoma [32]. Therefore, these results do not support an increased reliance on glycolysis in wasted SkM, despite lower density of functional mitochondria. Taken together, data suggest that anticancer drugs promote the accumulation of dysfunctional mitochondria in SkM and an overall decrease in its metabolic rate characterized by decreased oxidative capacity, glycolysis, and lipid accumulation.

#### 3.2.2. IGF-1/PI3K/Akt/mTOR Pathway

The IGF-1/PI3K/Akt/mTOR pathway is known to stimulate protein synthesis in SkM [10,106,107]. This signaling mechanism is downregulated by most of the cytotoxic agents used in chemotherapy. Cisplatin plus 5-FU plus leucovorin was shown to reduce the levels of the downstream target of the IRS-1 [88] and doxorubicin downregulated IRS-1 expression [17], whereas IGF-1, the triggering player of this pathway, was reported to be diminished by cisplatin [21]. The downregulation of the downstream player Akt was observed in some animal studies using different anticancer drugs [21,27,31] and in in vitro studies [88,89], resulting in reduced phosphorylation of mTOR and, eventually, of p70S6 kinase (p70S6K) and eukaryotic translation-initiation factor 4E binding protein 1 (4E-BP1). Indeed, decreased phosphorylation of these proteins was observed in L6 skeletal myoblasts [88]. mTOR may regulate SkM mass through two complexes, the mTOR complex 1 and complex 2 (mTORC1 and mTORC2) [10]. mTORC1 inhibits 4E-BP1 and activates p70S6K, enhancing protein synthesis, whereas mTORC2 phosphorylates Akt at serine 473, which was reported to be diminished in mice *soleus* and EDL [27]. In addition, mTORC2 induces autophagy through FOXO3 activation [10,106]. Overall, such data highlight the downregulation of IGF-1/PI3K/Akt/mTOR pathway in SkM following treatment with anticancer drugs.

#### 3.2.3. Regulation of Satellite-Cell Activation

Satellite cells are a heterogeneous group of stem cells [108] that mediate the life-long maintenance of SkM tissue [109]. These cells are responsible for postnatal muscle growth, repair, and regeneration [110], and are fundamental for SkM function. In atrophic conditions, a reduction of satellite-cell number and differentiation capacity was reported, thus affecting SkM regeneration [111]. Both MYF5 and Pax7 satellite cells are fundamental players in SkM regeneration [111]. MYF5 is a myogenic factor essential to muscle development and regeneration because it plays an important role in myogenic differentiation and maintenance [112]. Similarly, Pax7 is a key transcriptional regulator in SkM that, when activated, induces proliferating myogenic precursor-cell differentiation [113]. Curiously, an increase in MYF5 content was reported in mouse *soleus*, whereas in EDL, the Pax7^+^ was decreased following treatment with doxorubicin [18]. Therefore, the role of anticancer drugs on the regulation of satellite-cell activation needs to be further explored.

There are several pathways that modulate the satellite-cell maturation process. In fact, a JAK/STAT signaling cascade has been suggested to be required for the regeneration of muscle fibers [97], but upregulation of JAK/STAT signaling was reported to inhibit satellite-cell function [114]. IGF-1/PI3K/Akt/mTOR pathway may also stimulate satellite-cell differentiation acting through many steps of the process. For example, IGF-1 may stimulate satellite-cell proliferation and differentiation [115], whereas protein synthesis induced by the IGF-1/PI3K/Akt/mTOR pathway contributes to the maturation of myotubes, thus leading to muscle-fiber regeneration [111]. However, as previously described, cisplatin diminished IGF-1 expression [21] and the IGF-1/PI3K/Akt/mTOR pathway was reported to be downregulated [21].

#### 3.2.4. Myostatin/ActRIIB Pathway

**Figure 1:**
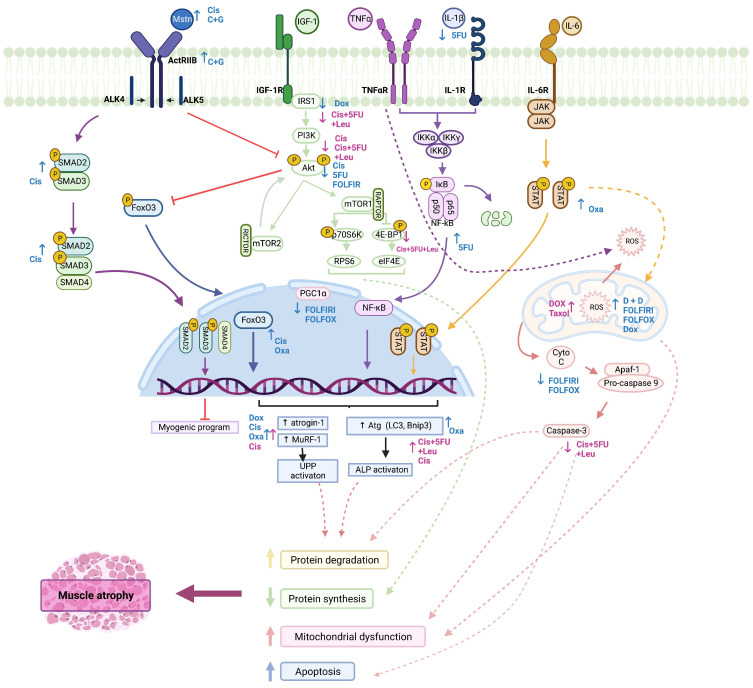
Signaling pathways modulated by chemotherapy in skeletal muscle. Doxorubicin decreases IRS1, NF-κB, and PGC1α expression, which consequently increases the expression of UPP (atrogin-1 and MuRF-1 ligases), and in mitochondria increases ROS generation. ROS levels also increased following doxorubicin plus dexamethasone treatment. Cisplatin increases phosphorylation of SMAD2, Mstn, and FOXO3 and induces atrogin-1 and MuRF-1 mRNA expression, thereby reducing Akt levels. When combined with gemcitabine, besides up-regulating atrogin-1, MuRF-1, Mstn, and FOXO3, cisplatin also stimulates ActRIIB. Oxaliplatin enhances STAT phosphorylation and activates both UPP and APL by increasing the expression of atrogin-1, MuRF-1, and Atg genes such as LC3 and BNIP3. 5-fluorouracil diminishes Akt levels and increases both p38 phosphorylation and NF-κB levels. FOLFIRI and FOLFOX act similarly, both decreasing PGC1-α protein levels and increasing ROS levels; however, FOLFIRI also reduces Akt levels. Legend: Cis: cisplatin; Dox: doxorubicin; C+G: cisplatin plus gemcitabine; Oxa: oxaliplatin; D+D: doxorubicin plus dexamethasone; Cis+5FU+Leu: cisplatin plus 5-fluorouracil plus leucovorin. Blue: modulation of animal studies; pink: modulation of in vitro studies. Created with BioRender.com (accessed on 16 February 2023).

The growth-differentiation factor 8 (GDF-8), also known as myostatin, is an autocrine/paracrine cytokine and a member of the TGF-β family. This cytokine is highly expressed in SkM compared with cardiac muscle or adipose tissue and it negatively regulates SkM mass and growth [10,116,117]. Myostatin signaling is modulated by anticancer drugs. Indeed, myostatin mRNA was raised in *quadriceps* treated with cisplatin [21] and *gastrocnemius* and *soleus* muscles treated with gemcitabine [29], whereas the expression of activin A, another member of the TGF-β superfamily, was increased in *gastrocnemius* and *soleus* muscles after gemcitabine therapy [29]. When activated, myostatin binds to the ActRIIB, which was reported to be increased in female-mouse SkM after treatment with gemcitabine [29]. The myostatin/ActRIIB complex leads to the activation of receptor-like kinase 4 or 5 (ALK4 and ALK5) [10,116,117]. The complex formed activates transcription factors SMAD2 and SMAD3 through phosphorylation [10,116,117]. Cisplatin increases SMAD2 phosphorylation [21]. These two members of the SMAD family form a trimeric complex with SMAD4 that can translocate into the nucleus and activate or inhibit the transcription of genes, such as the one for myoblast-determination protein 1 (MyoD), inhibiting the myogenic program (Figure 1) [10,116,117]. In general, the data imply that anticancer drugs contribute to diminished muscle growth and differentiation by raising myostatin expression.

#### 3.2.5. IL-6/JAK/STAT Pathway

The JAK/STAT pathway mediates the effect of diverse cytokines, such as IL-6. Although the levels of IL-6 were reported to be unchanged in different muscles [17,26], this cytokine binds to its receptor complex, IL-6R-gp130, activating the tyrosine kinase JAK [97]. Activated JAK undergoes a conformational change characterized by dimerization and phosphorylation and then activates the signal transducer and activator of transcription proteins [97]. Indeed, oxaliplatin seems to activate STAT3 phosphorylation [23], thus promoting its translocation to the nucleus and binding to specific regulators, enhancing the protein-coding genes in the promoter region, such as the E3 ligases muscle atrophy F-Box protein 32 (MAFbx/atrogin-1) and muscle RING-finger 1 (MuRF-1) [97]. The data indicate that anticancer drugs promote the activation of JAK/STAT pathways, boosting proteolysis by enhancing atrogin-1 and MuRF-1.

#### 3.2.6. NF-κB Pathway

NF-κB activation in SkM is triggered by diverse stimuli that are often related to inflammation, such as proinflammatory-cytokine interleukin 1 (IL-1) and tumor-necrosis-factor alpha (TNF-α). Even though the NF-κB pathway is responsible for regulating proinflammatory-cytokine production, leukocyte recruitment, or cell survival, these functions can either protect against inflammation or enhance it. This transcription factor is dimeric and can form homo- or heterodimers of distinct subunits, such as p65, p50, and p100, among others. These dimers are often activated in wasting conditions [10,118]. When stimulated, NF-κB can act through two different routes, the canonical and the non-canonical pathways, the first of which is responsible for survival, proliferation, inflammation, and immune regulation. This pathway involves the activation of p50/p65 dimers, which translocate into the nucleus, where it binds to the appropriate cognate DNA-binding sites, thus inducing diverse gene transcriptions such as those belonging to UPP and ALP (Figure 1) [10,118]. Indeed, 5-FU treatment increased phosphorylation of the p65 subunit in *soleus* and EDL [27]. When TNF-α binds to its receptor, it activates several intermediary steps that result in the activation of a complex composed of two catalytic subunits, IKK-α and IKKβ, and a regulatory subunit, IKK-γ/NF-κB essential modulator (NEMO) [10,118]. Curiously, unchanged TNF-α mRNA was reported after doxorubicin [17] and 5-FU [26] treatment, whereas decreased interleukin 1 beta (IL-1β) mRNA was observed in male mice treated with 5-FU [26].

#### 3.2.7. Autophagy–Lysosome Pathway

The ALP refers to a fundamental process for the removal of misfolded proteins and damaged organelles. The process prevents the accumulation of protein aggregates, thus maintaining protein homeostasis in SkM [10,95]. Several players of this pathway have been evaluated in SkM to mechanistically explain chemotherapy-induced muscle wasting, namely, the lipidated microtubule-associated protein 1 light-chain 3 alpha (LC3), p62 [10,95], and BNIP3 [111]. Indeed, BNIP3 mRNA was increased following treatment with oxaliplatin in male mice [23], as well as in C2C12 mouse myoblasts in the presence of cisplatin [89]. Other in vitro studies (Table A1) also supported the activation of the ALP pathway induced by anticancer drugs since LC3CII mRNA [89] and beclin1 mRNA [88] were increased. Several components of other pathways, such as AMPK, mTOR, mitochondrial proteins (OPA1, DRP1), and NF-κB, can mediate autophagy, thus activating or inhibiting ALP [10,119]. In fact, AMPK phosphorylated at tyrosine 172 levels increased in EDL after doxorubicin [17], which may activate autophagy at different regulation levels by phosphorylating specific ALP-related complexes, including ULK1 and PIK3C3/VPS34 [120].

#### 3.2.8. Ubiquitin–Proteasome Pathway

UPP is one of the main proteolytic systems responsible for the degradation of misfolded or defective proteins in SkM [10,95,121]. This pathway involves ubiquitin-activating enzymes (E1), ubiquitin-conjugating proteins (E2s), and ubiquitin–protein ligases (E3s), which act sequentially to polyubiquitinate the proteins that are then recognized by the 26S proteasome [10,121]. In SkM, MuRF-1 and atrogin-1 are two muscle-specific E3 ubiquitin ligases involved in UPP-mediated proteolysis. Both MuRF-1 and atrogin-1 are usually upregulated in atrophic muscle, as they are considered markers of muscle wasting [10,95,121]. Nevertheless, MuRF-1 and atrogin-1 mRNA were unchanged after FOLFIRI and FOLFOX treatment [31] and, unexpectedly, MuRF-1 mRNA decreased in male mice treated with cisplatin [20]. Still, MuRF-1 and atrogin-1 mRNA were increased by distinct chemotherapy agents in several animal studies ([13,21,23,29]) and one in vitro study [89]. These inconsistent results about MuRF-1 and atrogin-1 expression among studies may be explained by the differences in the chemotherapy agents, drug doses, animal species, or health status.

Activation of FOXOs family members such as forkhead box O3 (FOXO3) can also lead to activation of atrogin-1 and MuRF-1, thus increasing UPP activity. FOXO3 mRNA was enhanced in animals submitted to gemcitabine plus cisplatin [29] and to oxaliplatin [23]. Similarly, cisplatin increased FOXO3 mRNA but decreased FOXO3a phosphorylation in *quadriceps* muscles [21], enhancing the activation of the UPP by increasing the expression of both atrogin-1 and MuRF-1. Another FOXO element, FOXO1, was evaluated following anticancer treatment. However, FOXO1 mRNA was unchanged [21,23].

Taken together, anticancer drugs contribute to the activation of several pathways, such as NF-κB, IL-6/JAK/STAT, as well as directly increasing expression of the two muscle-specific E3 ubiquitin ligases, thus leading to enlarged UPP activity.

## 4. Conclusions

In this narrative review, we analyzed the molecular effects of chemotherapy agents on SkM based on the existing literature. The effects of cystemustine, gemcitabine, docetaxel, paclitaxel, and other chemotherapy agents on SkM have been studied; however, the number of studies is small when compared with those on doxorubicin, cisplatin, and 5-FU. Doxorubicin, one of the most studied chemotherapeutic agents, is very effective against cancer but its use is limited by dose-dependent toxic side effects in many organs, such as the heart and SkM. Therefore, studying the effects of other existing therapeutic regimens, including newer ones, such as the combination of 5-FU, leucovorin, oxaliplatin, and docetaxel (FLOT), is critical to better understanding the systemic changes induced by therapy and to explaining the associated side effects. One of the major limitations of clinical trials is the ethical constrains on the collection of SkM biopsies from cancer patients, although these samples are essential for a better understanding of the cellular and molecular changes induced by chemotherapy in SkM. Another limitation in the investigation of this issue is the low number of preclinical studies investigating this issue, with only three studies having been performed with C2C12 or L6 cell lines so far. To improve the translational application of preclinical research, human cell lines, such as the RCMH cell line, should be considered [122].

In addition, more preclinical studies using tumor-bearing animals and clinical trials with well-defined criteria are needed to support the development of novel pharmacological and nonpharmacological therapies. Such criteria should include the chemotherapy regimen, cancer stage, number of chemotherapy cycles, and duration of treatment. By integrating pharmacological therapies with exercise training, nutrition, and psychological support into tailored multimodal approaches, cancer patients at risk of muscle wasting can achieve better postoperative recovery. Therefore, it is crucial to prioritize research efforts in this area to improve the quality of life of cancer patients and enhance survival outcomes.

## Data Availability

Not applicable.

## Appendix A

**Table A1:** In vitro and ex vivo studies about the effects of chemotherapy.

Chemotherapy	Sample	Main Results	Ref.
Doxorubicin 50 or 175 μM Incubated for (0–120 min)	Male CD2F1 mice, EDL muscle (ex vivo)	↓ Maximal forces, maximal relaxation, maximal contraction velocity	[87]
Cisplatin 10, 50, or 100 μM Incubated for up to 72 h	C2C12 myoblasts	↓ Size of myotubes (50%), Cav-3, myogenin, and p-Akt levels ↑ Atrogin-1, BNIP3, and LC3-II expression	[89]
Taxol (40 nM), doxorubicin (0.2 μM), or cisplatin (10 μM) Incubated over 3 days	C2C12 myoblasts	Taxol: ↑ Tubulin and detyrosinated tubulin expression, ROS production ↓ Myotube myosin content, mitochondrial content, Prx 3 expression	[58]
		Doxorubicin:↑ oxidized Prx 3, ROS production ↓ Mitochondrial content, myotube diameter	
		Cisplatin: ↓ myotube myosin content; = ROS production	
Cisplatin (20 μg/mL), 5-fluorouracil (50 μg/mL), and leucovorin (10 μg/mL) Incubated for 24 h, 48 h, or 72 h	L6 myoblasts	↓ Peptide chain initiation in myotubes, myofibrillar, and sarcoplasmic fractions; p-AKTSer473, p-S6Ser235/236, p-S6K1Thr389, p-4EBP1Thr37/46, p-IRS1 (60%), GSSer641 (60%), IRS-1 level, caspase 3 (50%), caspase 7 (tend to), p62 abundance in myotubes (48 h), SDHA (50%-60%) and COXIV (75%) levels, and amino-acid transporter SLC38A9/SNAT1 (>50%) ↑ p-GS (50%), IRS1Ser612(70%), beclin1 (tend to), LC3BII, cleaved caspase 3 = Cleaved caspase 7	[88]

↑ increase; ↓ decrease; = no change. Abbreviations: Akt: protein kinase B; Cav-3: caveolin 3; COXIV: cytochrome oxide; EDL: extensor digitorum longus; IRS-1: insulin-receptor substrate 1; LC3-II: LC3-phosphatidylethanolamine conjugate; P-GS: cyclooxygenase; Prx 3: peroxisome assembly factor; ROS: reactive oxygen species; SDHA: succinate dehydrogenase; SkM: skeletal muscle; SNAT1: sodium-coupled neutral amino-acid transporter 1.
